# Supplementary material for: Exploring Mechanisms of Antifungal Lipopeptide Iturin A from Bacillus against Aspergillus niger
Source: J Fungi (Basel). 2024 Feb 24;10(3):172. doi: 10.3390/jof10030172 (PMC10970988; doi:10.3390/jof10030172)
Supplement: Supplementary file 1 [file jof-10-00172-s001.zip › jof-2871652-Supplementary.pdf]

Labelfree Proteomics Assay Reference Methods

1. Materials and Reagents

TableS1: Main Materials and Reagents

Reagent name	Supplier
trypsin	Promega
acetonitrile	Fisher Chemical
trifluoroacetic acid	Sigma Aldrich
formic acid	Fluka
iodoacetamide	Sigma
dithiothreitol	Sigma
urea	Sigma
trichloroacetic acid	Sigma
protease inhibitor	Calbiochem
EDTA	Sigma
TEAB	Sigma
H_2O	Fisher Chemical

TableS2: Instrument list

Instrument	Model	Brand
Ultra High Performance Liquid Chromatograph	EASY-nLC 1200	Thermo Fisher Scientific
High Resolution Mass Spectrometry	Q-Exactive HF	Thermo Fisher Scientific

2. Methods

2.1. Protein Extraction

Suitable protein extraction and precipitation purification methods were selected based on the samples, and the protein concentration of each sample was determined using the kit.

2.2. Enzymatic Desalination

An appropriate amount of total protein was taken and added to the reducing agent buffer and reacted for 1 hour at 37°C. The reaction was carried out in the dark for 45 min at room temperature. Subsequently, IAA was added to a final concentration of 50 mM and the reaction was carried out in the dark for 45 min at room temperature. The reaction was quenched by the addition of 1M DTT solution of IAA, and the final concentration of urea was brought to less than 1 mol/L using 50 mmol/L NH_4HCO_3 solution. Finally, sequencing grade trypsin solution was added, incubated overnight at 37°C, and the digested peptides were desalted using a C18 solid phase extraction column.

2.3. LC-MS Analysis

Mobile phase A was an aqueous solution containing 0.1% (v/v) formic acid; mobile phase B was acetonitrile containing 0.1% (v/v) formic acid. Peptides were dissolved in phase A of the liquid chromatography mobile phase and separated using

the nanoACQUITY UPLC M-Class system (Waters, USA). The liquid gradient was set from 0 to 120 min, 8% to 100% mobile phase B. The flow rate was maintained at 300 nL/min.

The peptides were separated by the ultra-high performance liquid phase system and injected into the NSI ion source for ionization, and then analyzed by the Q Exactive HF mass spectrometer. The ion source voltage was set to 2.3 kV, and both the peptide precursor ions and their secondary fragments were detected and analyzed using a high-resolution Orbitrap in the Q Exactive HF. The scanning range of the primary mass spectrometer was set to 400-1800 m/z, the scanning resolution was set to 60000, and the secondary scanning resolution was set to 15000. The data acquisition mode uses a data-dependent (DDA) program, that is, after the first-level scan, the precursor ions of the top 20 peptides with the highest signal intensity are selected and sequentially entered into the HCD collision cell for fragmentation with a fragmentation energy of 28ev, and the second-level mass spectrometry is also performed sequentially. To improve the effective utilization of mass spectrometry, the automatic gain control (AGC) was set to 3E6, the signal threshold was set to 10000 ions, the maximum ion injection time was set to 50 ms, and the dynamic exclusion time of tandem mass spectrometry scanning was set to 45 s to avoid repeated scans of precursor ions.

TableS3: LC mobile phase conditions

**Time(min)**	**Flow rate(μL/min)**	**A%**	**B%**
0	300	92	8
98	300	72	28
113	300	63	37
117	300	0	100
120	300	0	100
